# Respiratory Mucosal Immunity: Kinetics of Secretory Immunoglobulin A in Sputum and Throat Swabs From COVID-19 Patients and Vaccine Recipients

**DOI:** 10.3389/fmicb.2022.782421

**Published:** 2022-02-25

**Authors:** Cuiping Ren, Yong Gao, Cong Zhang, Chang Zhou, Ying Hong, Mingsheng Qu, Zhirong Zhao, Yinan Du, Li Yang, Boyu Liu, Siying Wang, Mingfeng Han, Yuxian Shen, Yan Liu

**Affiliations:** ^1^Department of Microbiology and Parasitology, Anhui Medical University, Hefei, China; ^2^Anhui Provincial Laboratory of Pathogen Biology, Anhui Medical University, Hefei, China; ^3^Anhui Key Laboratory of Zoonosis of High Institution, Anhui Medical University, Hefei, China; ^4^Laboratory of Tropical and Parasitic Diseases Control, Anhui Medical University, Hefei, China; ^5^School of Basic Medical Sciences, Anhui Medical University, Hefei, China; ^6^Department of Clinical Laboratory, The Second People’s Hospital of Fuyang, Fuyang, China; ^7^Maanshan Center for Disease Control and Prevention, Maanshan, China

**Keywords:** COVID-19, mucosal immunity, IgA, sputum, throat swab, nucleocapsid protein, vaccine

## Abstract

While IgM and IgG response to SARS-CoV-2 has been extensively studied, relatively little is known about secretory IgA (sIgA) response in respiratory mucosa. Here we report IgA response to the SARS-CoV-2 in sputum, throat swabs, and serum with nucleocapsid protein (NP) enzyme-linked immunosorbent assays (ELISA) in a cohort of 28 COVID-19 patients and 55 vaccine recipients. The assays showed sIgA in respiratory mucosa could be detected on the first day after illness onset (AIO), and the median conversion time for sIgA in sputum, throat swabs, and serum was 3, 4, and 10 days, respectively. The positive rates of sIgA first week AIO were 100% (24/28) and 85.7% (24/28) in sputum and throat swabs, respectively, and were both 100% during the mid-onset (2–3 weeks AIO). During the recovery period, sIgA positive rates in sputum and throat swabs gradually decreased from 60.7% (17/28) and 57.1% (16/28) 1 month AIO and the sIgA antibodies were all undetectable 6 months AIO. However, serum IgA positive rate was still 100% at 4 months and 53.6% (15/28) at 6 months. Throat swabs obtained from volunteers who received inactivated SARS-CoV-2 vaccines by intramuscular delivery all showed negative results in IgA ELISA. These findings will likely improve our understanding of respiratory mucosal immunity of this emerging disease and help in containing the pandemic and developing vaccines.

## Introduction

Severe acute respiratory syndrome coronavirus 2 (SARS-CoV-2) virus is very infectious, primarily infecting the respiratory tract mucosal surfaces (Gorbalenya et al., 2020; Zhu et al., 2020; Hu et al., 2021). Mucosal immunity in the upper airways and nasal passages is particularly important as the first defensive barrier, affecting the initial viral spread (Mazanec et al., 1995; See et al., 2006). Among antibody isotypes, secretory immunoglobulin A (sIgA) at mucosal surfaces plays a crucial role in protecting against respiratory virus infection (Mazanec et al., 1995; Terauchi et al., 2018). A previous study by Sterlin et al. (2021) reported that both serum IgA and mucosal IgA could effectively neutralize SARS-CoV-2 and dominated the neutralizing antibody response to SARS-CoV-2 in the early phase of infection. Recent studies have reported that dimeric IgA, the secretory form of IgA in the mucosa, was a more potent neutralizer than IgG and serum IgA monomers against authentic SARS-CoV-2 (Ejemel et al., 2020; Wang et al., 2021). These results suggest that SARS-CoV-2 induces specific sIgA and strong mucosal immunity within the respiratory system effectively against virus infection.

It was already known that vaccines delivered by inhalation can elicit IgA response in both mucosal surfaces and serum, whereas vaccines delivered intramuscularly primarily elicit serum IgG (Torrieri-Dramard et al., 2011; Ku et al., 2021; Lund and Randall, 2021). van Doremalen et al. (2021) recently reported that intranasal ChAdOx1 nCoV-19/AZD1222 vaccination, an approved adenovirus-vectored vaccine, reduced shedding of SARS-CoV-2 from the upper respiratory tract in vaccinated macaques and hamsters, whereas intramuscular vaccination protected against lung inflammation and pathology but did not reduce shedding. A similar result was also reported by Hassan et al. (2020) adenovirus-vectored vaccine ChAd-SARS-CoV-2-S delivered by inhalation could provide more effective sterilizing protection than intramuscular delivery, and promote systemic and mucosal IgA response when intramuscular delivery failed to induce IgA.

Although IgA response to SARS-CoV-2 in respiratory tract is particularly important in neutralizing the virus and affecting the initial viral spread, long time longitudinal studies of mucosal IgA kinetics were relatively rarely reported. In this study, we established an indirect ELISA method for IgA detection using nucleocapsid protein (NP) and detected COVID-19 samples of patients collected at different time points within 6 months and throat swabs of 55 volunteers who have received at least one dose of inactivated SARS-CoV-2 vaccines. Our results showed the kinetics of sIgA to SARS-CoV-2 in sputum and throat swabs and non-secretory IgA in the blood sample, which could improve our understanding of the mucosal immune response of the virus and provide new ideas for immunological evaluation of pandemic prevention and control.

## Materials and Methods

### Clinical Specimens

Throat swabs, sputum, and serum were collected from 28 laboratory-confirmed COVID-19 patients who were hospitalized at the Second People’s Hospital of Fuyang in Anhui, China between February 8 and September 25, 2020. Sequential specimens at various time points after illness onset of these patients were collected for SARS-CoV-2 NP IgA ELISA. Thirty healthy subjects without any known history of SARS-CoV-2 infection were recruited, and throat swabs, sputum, and serum specimens were collected as healthy control. Serum specimens collected contemporaneously in 2020 from 30 human immunodeficiency virus (HIV)-positive patients, 30 hepatitis B (HB) patients, and 30 hepatitis C (HC) patients and throat swabs from 30 unknown fever (UF) and 30 patients with H1 influenza were provided by Maanshan Municipal Center for Disease Control and prevention. In this study, we also obtained throat swabs from 55 vaccinated volunteers who received at least one dose of inactivated SARS-CoV-2 vaccine (Sinovac Life Sciences) by intramuscular delivery for NP IgA detection. Throat swabs were collected with synthetic fiber swabs by physicians and inserted into 3-ml viral transport medium. Sputum samples were collected in a 50-ml screw-top plastic tube containing 3 ml of viral transport medium. All samples were stored at −80°C since collection. Detailed information of the COVID-19 patients and vaccinated volunteers are shown in Supplementary Table 1.

Informed consent was obtained from all individual participants included in the study. The study was approved by the Ethics Committee of Anhui Medical University, with adherence to the Declaration of Helsinki. The approval ID of Ethics Committee is 2020H015.

### Severe Acute Respiratory Syndrome Coronavirus 2 Nucleocapsid Protein Immunoglobulin A Enzyme-Linked Immunosorbent Assays

SARS-CoV-2 NP IgA ELISA was performed according to the conventional method (Ren et al., 2017). The NP antigen used is a recombinant eukaryotic expression protein. It was purchased from T&J Biomedical, Beijing, China. The HRP-labeled rabbit anti-human IgA was purchased from Abcam, Cambridge, United Kingdom (ab97215). The operational concentrations of clinical samples, antigen, antibodies, and reaction time were determined in preliminary experiments using the chessboard method. Mixed serum samples of 30 individuals with SARS-CoV-2 infection and mixed serum samples from 30 healthy subjects were used in preliminary experiments.

Based on the results of the preliminary experiments, the SARS-CoV-2 NP IgA ELISA was established. The microtiter plates were coated with 100 μl/well of NP antigen (5 μg/ml), overnight at 4°C. After washing three times with 0.05% Tween20-PBS (w/v) and blocking with 2% BSA-PBS (w/v) for 1 h, the plates were incubated with specimens for 1 h. Serum was diluted at 1:200 and added 100 μl/well. For each sputum or throat swab specimen, 100 μl/well was added. Samples had been heated at 56°C for 30 min to inactivate the virus before the SARS-CoV-2 NP IgA ELISA experiments. The HRP-labeled rabbit anti-human IgA (Abcam, United States) was diluted 1:100,000. All steps were carried out at room temperature. Absorbance values were read using a plate spectrophotometer (Molecular Devices, United States) at a wavelength of 450 nm. Positive and negative controls were used throughout the study. A blank was also included on each plate. Each sample was tested in duplicate. The cutoff value for a positive reaction was the mean plus two standard deviations of the absorbance reading in the controls, and it was 0.235 in our SARS-CoV-2 NP IgA ELISA.

### Statistical Analysis

The OD_450_ values of sputum, throat swab, and serum detected by IgA ELISA at different time points were statistically analyzed by repeated measures ANOVA or multiple independent sample non-parametric test. The positive rates of IgA ELISA in serum, throat swab, and sputum were statistically compared with the Chi-square test or Fisher’s exact test. Power calculation has been done. The sample size of the current study may not be optimal, but should be sufficient to draw a conclusion that may guide clinical practice. A *p*-value < 0.05 was considered statistically significant. All statistical analyses were conducted by SPSS software.

## Results

### Severe Acute Respiratory Syndrome Coronavirus 2 Nucleocapsid Protein Immunoglobulin A Enzyme-Linked Immunosorbent Assays

**FIGURE 1:**
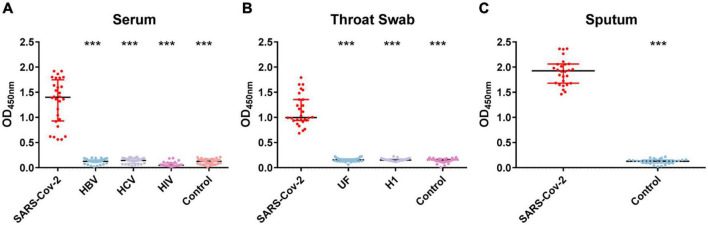
OD_450_ value of nucleocapsid protein (NP)–immunoglobulin A (IgA) enzyme-linked immunosorbent assay (ELISA) for the detection of the NP-IgA antibody from non-SARS-CoV-2 infected patients and severe acute respiratory syndrome coronavirus 2 (SARS-CoV-2)-infected patients. **(A)** Serum samples from different populations; HBV represents patients with hepatitis B virus infection, HCV represents patients with hepatitis C virus infection, HIV represents HIV-positive patients. **(B)** Throat swab samples from different populations; UF represents patients with unknown fever, H1 represents patients with H1 influenza. **(C)** Sputum samples from SARS-CoV-2-infected patients and healthy controls. Data are presented as the median with interquartile range. ***p < 0.001 (two-tailed multiple comparison test with Kruskal–Wallis method).

To evaluate the sensitivity of COVID-19 NP-IgA ELISA, the levels of SARS-CoV-2-specific NP-IgA antibodies were measured in sputum (*n* = 28), throat swabs (*n* = 28), and serum specimens (*n* = 28) of COVID-19 patients 15–21 days after infection. The results showed that the positive rates of antibody in sputum, throat swabs, and serum were all 100% (Figure 1). No cross-reactivity was found in the serum collected from patients uninfected with SARS-CoV-2 but diagnosed with hepatitis B (HB, *n* = 30), hepatitis C (HC, *n* = 30), or HIV infection (*n* = 30) detected by SARS-CoV-2 NP IgA ELISA. In addition, there was no positive reaction in the throat swabs of flu patients (*n* = 30) and unknown fever (UF, *n* = 30). None of the healthy volunteers, including serum specimens (*n* = 30), sputum samples (*n* = 30), and throat swabs (*n* = 30), tested positive for IgA (Figure 1). The assay showed an overall specificity of 100%.

### Nucleocapsid Protein Immunoglobulin A Detection in COVID-19 Patients and Vaccinated Volunteers

Using the method of NP IgA ELISA we established, we detected NP IgA levels of serum, throat swabs, and sputum sequential samples of 28 COVID-19 patients and throat swabs of 55 vaccinated volunteers.

**FIGURE 2:**
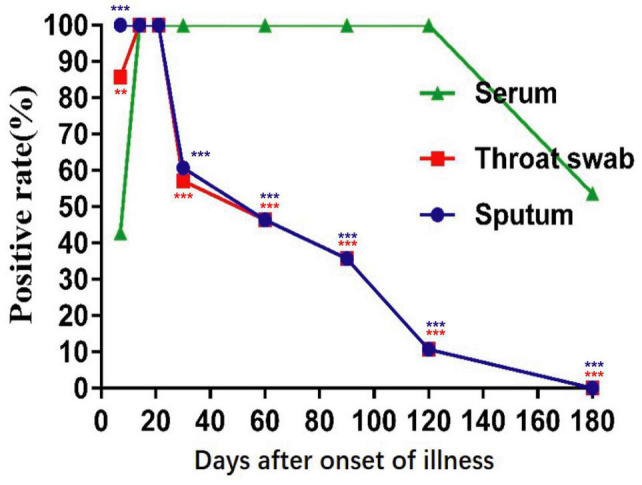
Positive rates of NP-IgA ELISA for detection of the sequential samples of 28 COVID-19 patients with different disease courses from the symptom onset. Sequential samples include serum, throat swabs, and sputum from all phases of the disease. **p < 0.01, ***p < 0.001 (compared with positive rates of serum, two-tailed Fisher’s exact test).

**FIGURE 3:**
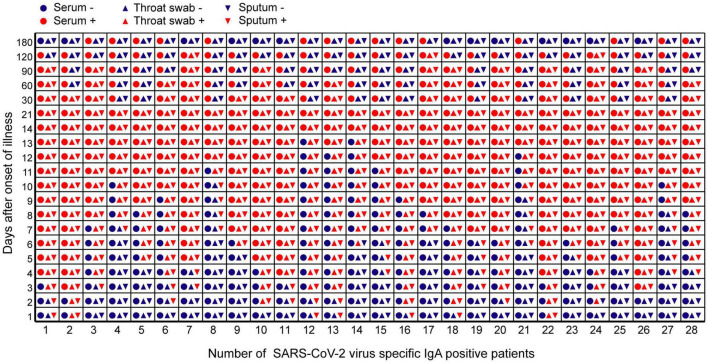
The time point of positive or negative results of IgA antibody against SARS-CoV-2 in different samples of 28 COVID-19 patients during the observation period. The abscissa represented the number of 28 COVID-19 patients during the observation period. The ordinate represented days after symptom onset. Red icons meant that IgA antibody test results are positive. Blue icons meant that IgA antibody turns negative.

**FIGURE 4:**
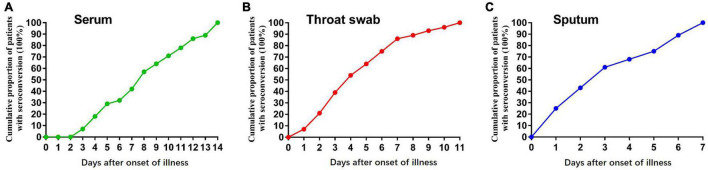
Graph of positive rates of SARS-CoV-2 virus-specific IgA vs. days after symptom onset in the sequential samples of 28 COVID-19 patients. **(A)** Serum samples, **(B)** Throat swab samples, and **(C)** sputum samples from 28 COVID-19 patients.

**FIGURE 5:**
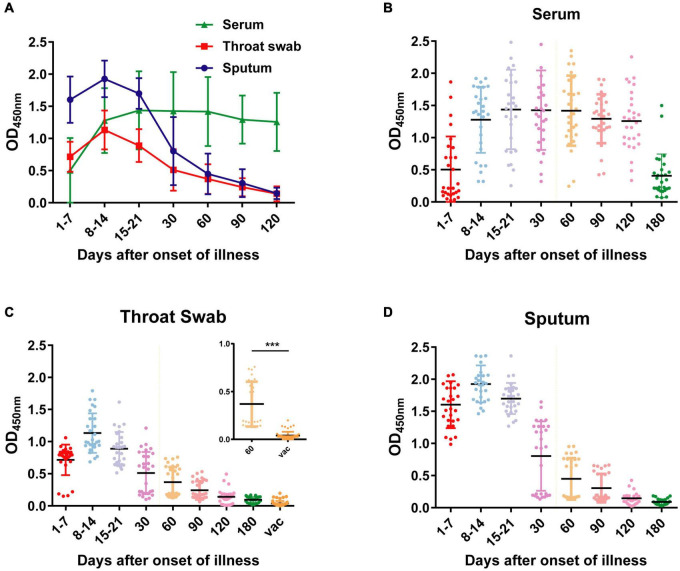
OD_450_ values of NP-IgA ELISA for detection of the sequential samples of 28 COVID-19 patients and 55 vaccine recipients. **(A)** OD_450_ values of the sequential serum, throat swab and sputum samples of 28 COVID-19 patients. Scatter plots of OD_450_ values of **(B)** serum samples, **(C)** throat swab samples, and **(D)** sputum samples at different phases of the disease or 50 d after vaccine. Vac, represents vaccine recipients. Data are presented as the mean ± SD. ***p < 0.001 (two-tailed unpaired *t*-test).

In the early stages of infection, within 1 week after symptom onset, the NP IgA antibody positive rates of the sputum, throat swab, and serum specimens were 100% (28/28), 85.7% (24/28), and 42.9% (13/28), respectively (Figure 2). The positive rates of NP IgA antibody in sputum and in throat swabs were significantly higher than that in serum (Fisher’s exact test, throat swab vs. serum, *p* < 0.01, sputum vs. serum, *p* < 0.001). The NP IgA antibody of seven sputum specimens and two throat swab specimens among 28 patients could be detected on the first day of illness onset (Figure 3), while IgA in the serum was first detected on the third day after illness onset (Figure 3). According to the cumulative seroconversion curve, the median conversion time for IgA in sputum, throat swabs, and serum was 3, 4, and 10 days, respectively (Figure 4). The IgA positivity rates in three specimens were all 100% (28/28) from 2 to 3 weeks after symptoms (Figure 2). The OD_450_ value showed the highest IgA antibody titer in sputum and throat swabs for this time period (Figure 5A). These results indicate that there is an early IgA response in mucosal surfaces, which may particularly be important in neutralizing the virus at the upper respiratory tract and lungs.

During the recovery phase, the positive rates and OD_450_ value of NP IgA antibody in sputum and in throat swabs decreased significantly from 1 month after the onset of the disease (Figures 2, 5). The positive rates of IgA in sputum, and throat swabs were 60.7% (17/28) and 57.1% (16/28) at 1 month after illness onset, respectively, and both were significantly lower than that of the rate in serum 100% (28/28) (Figure 2) (Fisher’s exact test, throat swab/sputum vs. serum, *p* < 0.001). At 4 months after the onset of the disease, the rates dropped to 10.7% (3/28) in either sputum or throat swabs. The serum IgA positivity rate in COVID-19 patients remained 100% at 4 months after onset, and there is also no tendency for OD_450_ values to decrease (Figures 2, 5). After 6 months of the onset, the IgA antibody level in sputum and throat swabs of all COVID-19 patients were undetectable (Figure 2). The IgA positivity rate in the serum of patients dropped to 53.6% (15/28) (Figure 2). Our results suggested that the production of sIgA in the lung epithelium is time limited.

To evaluate the mucosal IgA level of vaccinated volunteers, we also obtained 60 throat swabs from 55 vaccinated volunteers at 50 days after vaccination. All the volunteers received at least one dose of inactivated SARS-CoV-2 vaccine by intramuscular delivery. Compared with the throat swab specimens of COVID-19 patients 60 days after illness onset, specimens from vaccinated volunteers had significantly lower OD_450_ value of detected IgA (Figure 5C) (two-tailed unpaired *t*-test, *p* < 0.001). All the throat swab specimens from vaccinated volunteers showed negative results, indicating insufficient mucosal immunity and IgA response in respiratory tract after the vaccination.

## Discussion

As a mucosal targeted virus, SARS-CoV-2 can generate sIgA and induce strong mucosal immunity (Arakawa et al., 2019; Yu et al., 2020). Our study reports the kinetics of sIgA response to SARS-CoV-2 in the mucosa of the respiratory system and non-secretory IgA in the blood. We found that IgA could be detected at the early stage of virus infection in sputum and throat swabs, and some patients even on the first day after the onset. The median conversion time for IgA in sputum and throat swabs was less than 4 days, 6–7 days earlier than in serum. Moreover, IgA in sputum could be all detected among patients within 1 week after the onset of the disease. Other studies have shown that the first seroconversion day of IgM and IgG in serum was 5 days after onset. The median conversion time for IgM and IgG was 14 and 14–15 days, respectively (Wang et al., 2020; Yu et al., 2020). The proportion of patients with positive virus-specific IgG reached 100%, approximately 17–19 days after symptom onset, while the proportion of patients with positive virus-specific IgM reached a peak of 94.1%, approximately 20–22 days after symptom onset (Long et al., 2020; Zhou et al., 2021). Our study demonstrated that IgA response to SARS-CoV-2 in the respiratory mucosa was much earlier than IgG and IgM. Other studies have reported that mucosal IgA had more efficient neutralization potential than IgG and was dominant in early SARS-CoV-2 neutralizing antibody (Sterlin et al., 2021; Wang et al., 2021). These results indicated particularly the important role of mucosal sIgA against the initial SARS-CoV-2 viral spread.

Meantime, our results suggested that there is a particular value of mucosal sIgA detection in COVID-19 diagnosis. Our SARS-CoV-2 NP IgA ELISA method showed excellent specificity in the detection of SARS-CoV-2-infected patients. There was no cross-reaction when it was used to detect other non-SARS-CoV-2-infected patients, such as HB, HC, HIV, UF, or H1 influenza. Our IgA ELISA assay could be useful when the suspected cases are repeatedly negative by RT-qPCR in the diagnosis of acute phase. Detection of sIgA can aid diagnosis as soon as possible, which is important for effective intervention and isolation of patients to prevent further spread of infection.

Moreover, according to our results, sIgA, compared with non-secretory IgA, would rapidly turn negative during the recovery period. NP IgA in the sputum and throat swab of the patient had turned negative partially at 1 month after onset and more than half by 2 months after onset. A previous study of Isho et al. (2020) detected anti-spike and anti-RBD IgA levels in the saliva of the patient, and the results also showed significant decreases from 1 month and barely detectable IgA level on day 100. These results indicated that production of sIgA in the lung epithelium is time limited. Although reinfection cases of SARS-CoV-2 was very uncommon, it occurred in 1.39% health-care workers at a median of 7 months after the onset of the first episode in a large, multicenter, prospective cohort study (Hall et al., 2021). Another report reviewed 60 cases of reinfection with viral sequencing, and episodes of infection were separated by a median of 116 days (Qureshi et al., 2021). As one crucial part of mucosal immunity barrier against virus invasion, the short duration of sIgA and insufficient level at an indicated time may contribute to the occurrence of reinfection.

Given that intramuscular vaccine primarily elicits serum IgG and IgM, the role of IgM and IgG neutralizing antibodies attract most attention of scientists. However, SARS-CoV-2 viremia is associated with COVID-19 severity, and viremia in patients generally occurred in severe illness (Jacobs et al., 2021; Li et al., 2021). Elicited IgM and IgG antibodies seem to mainly protect vaccinated people from severe pathology. Their antiviral effect on respiratory mucosal epithelial cells is not as important as secreted IgA, or it even has not much effect (Mazanec et al., 1995). Our study suggested that there is insufficient mucosal immunity and IgA response in respiratory tract in vaccinated people with intramuscular delivery. Despite various intramuscular delivery vaccines have shown high efficacy against SARS-CoV-2 infection, rare breakthrough infections have been reported, and most of them were mild or asymptomatic (Bergwerk et al., 2021). This may also be a problem that current vaccinators have to consider because even asymptomatic infected people could spread the virus and bring great challenge to the society. Interestingly, recent studies of two adenovirus-vectored vaccines showed that intranasal vaccination could reduce shedding of virus and induce higher levels of neutralizing antibodies, particularly systemic and mucosal IgA than intramuscular delivery (Hassan et al., 2020; van Doremalen et al., 2021). Therefore, to further improve the immune effect of the vaccine, multiple dosage forms of vaccine immunization strategies (injection and spray) should be considered by scientists and government decision-making departments.

A limitation of our study is that our results do not directly imply the protection of mucosal immunity against SARS-CoV-2, given that the NP antibody is not a neutralizing antibody. NP protein is a suitable candidate for diagnosis due to its high immunogenicity. Previous studies have shown closely similar patterns between anti-NP antibodies and anti-spike and anti-RBD IgA/IgG responses (Isho et al., 2020; Sterlin et al., 2021). Our study of kinetics of sIgA suggested that anti-NP IgA detection of sputum and throat swabs may have some diagnostic value for pandemic prevention and control.

## Data Availability Statement

The original contributions presented in the study are included in the article/Supplementary Material, further inquiries can be directed to the corresponding author/s.

## Ethics Statement

The studies involving human participants were reviewed and approved by the Ethics Committee of Anhui Medical University. The patients/participants provided their written informed consent to participate in this study.

## Author Contributions

CR, YG, and CZha carried out the experiment and drafted the manuscript. CZho, YH, MQ, ZZ, YD, LY, BL, and SW participated in the design and coordination of the study. YL, YS, and MH were the project coordinators, responsible for the project design, implementation, and oversaw all aspects of case definition, fieldwork, laboratory activities, and data analysis. All authors read and approved the final manuscript.

## Conflict of Interest

The authors declare that the research was conducted in the absence of any commercial or financial relationships that could be construed as a potential conflict of interest.

## Publisher’s Note

All claims expressed in this article are solely those of the authors and do not necessarily represent those of their affiliated organizations, or those of the publisher, the editors and the reviewers. Any product that may be evaluated in this article, or claim that may be made by its manufacturer, is not guaranteed or endorsed by the publisher.
