# Supplementary material for: Respiratory Mucosal Immunity: Kinetics of Secretory Immunoglobulin A in Sputum and Throat Swabs From COVID-19 Patients and Vaccine Recipients
Source: Front Microbiol. 2022 Feb 25;13:782421. doi: 10.3389/fmicb.2022.782421 (PMC8914317; doi:10.3389/fmicb.2022.782421)
Supplement: Supplementary file 1 [file Table_1.DOCX]

Table S1 Basic information of 28 COVID-19 patients included in the study

| Number | Sex | Age | Times((Positive nucleic acid test) | First-time clinical typing | Basic diseases |
| --- | --- | --- | --- | --- | --- |
| 1 | Male | 47 | 1 | ordinary case | No |
| 2 | Male | 55 | 1 | severe case | No |
| 3 | Female | 60 | 3 | ordinary case | Chronic gastric disease |
| 4 | Male | 56 | 1 | ordinary case | Hypertension |
| 5 | Male | 53 | 2 | ordinary case | Renal stone |
| 6 | Female | 52 | 1 | ordinary case | No |
| 7 | Male | 39 | 1 | ordinary case | No |
| 8 | Male | 47 | 1 | ordinary case | No |
| 9 | Male | 48 | 2 | ordinary case | No |
| 10 | Female | 21 | 2 | severe case | No |
| 11 | Female | 68 | 1 | ordinary case | Hypertension |
| 12 | Female | 68 | 1 | ordinary case | coronary artery disease |
| 13 | Female | 60 | 1 | ordinary case | No |
| 14 | Male | 40 | 1 | ordinary case | Hepatitis B cirrhosis |
| 15 | Female | 41 | 1 | Minor case | No |
| 16 | Male | 44 | 1 | ordinary case | Hepatitis B cirrhosis |
| 17 | Female | 63 | 1 | ordinary case | No |
| 18 | Male | 16 | 1 | Minor case | No |
| 19 | Female | 28 | 5 | Minor case | No |
| 20 | Male | 22 | 2 | ordinary case | No |
| 21 | Male | 50 | 1 | severe case | Hypertension |
| 22 | Male | 8 | 1 | Minor case | No |
| 23 | Female | 44 | 1 | ordinary case | No |
| 24 | Male | 26 | 2 | ordinary case | No |
| 25 | Female | 39 | 4 | ordinary case | Hypertension |
| 26 | Female | 36 | 1 | ordinary case | No |
| 27 | Female | 38 | 2 | ordinary case | No |
| 28 | Male | 32 | 1 | ordinary case | No |
